# Medical Science

**Published:** 1884-04

**Authors:** Edward B. Atkins


### MEDICAL SCIENCE.

I.

Of science now I fain would sing
And to her feet my tribute bring;
This is my muse—no idle theme,
No careless thought or fleeting dream:
Of queenly worth and merit true
The theme to which I ask your view,
While every student at her shrine
Shall find her laws almost divine;
Wilt thou, my muse, in song attend,
And now with her in beauty blend?

II.

The fall of man brought pain and death,
Their power latent in every breath;
Growth and decay, by nature's law,
Shape our being at every flaw;
The one to crush and back to earth
To carry our form, where we had birth;
The other to upbuild our frame,
And hold through life a form the same.

These are the laws by God designed,
Now acting through all life we find—
Here science shall unbroken reign
Till men the laws of life attain.

III.

In looking back o'er the ages dark
We find thy* light, a gleaming spark,
Adown the course of time to glow,
Till all our race thy blessings know.
Egyptian shade surrounds thy rise,
While modern age thy honors prize;
Disease and pain and injured part
Still tax thy skill or test thy art;
Thou child of nature and of time,
The gift of healing—*all* is thine,
No greater art can mortals know
Or greater boon than thine bestow.

IV.

He who first in thy pathway trod
Was known a man, was made—a God;
The homage of the Grecian mind,
That vainly sought thy source to find,
Made *Esculapius*, famed in song,
The source of health they worshiped long.
With God and nature both unknown,
They saw all power in man alone,
And, bowing low before their shrine,
*They* first made science *all* divine.

V.

But now how fallen from such high place
Are all thy sons in angry race!
Fair science hid in form unknown,
Where men pursue plans of their own;
'Tis sad to tell, and still too true,
Those who led thy advance were few;
He who improved the healing art
From his fellows was doomed to part.
The prestige of some noble name
Gave plans of treatment world-wide fame,
And he who would such plans displace
Was rudely driven from his place.

VI.

Physicians met in grave debate,
While death sealed their patients' fate,
Devising *codes* their hours employ;
Disease spreads on, life to destroy;
*Our Goddess* then dire discord saw,
Where men would narrow heaven's law,
And make the healing of our race
Subserve the strife for power's place.

---

* *Science.*

VII.

But looking *on*, her face grew bright,
Some she saw were schooled aright,
No narrow code could bind their view
Or make their minds as narrow too;
Wide as the wants of all our race
They saw their mission's godlike place,
Nor can the laws which men enact
From their powers of cure detract.

VIII.

Now sullen sons, who oft deride
All plans of good they have not tried,
In council deep, with angry frown,
Would every new advance put down—
Give to science her deepest grief
But fail to give their sick relief.

IX.

Then should some son, less wed to form,
New plans pursue, and such alarm,
He is at once a quack decreed,
And from him their robes are freed;
We search thy fields of learning through
And narrow minds oft come to view,
Some there are who but fault can see
'Mid all the good that e'er may be,
So in this realm of healing art
They fain would rend thy laws apart.

X.

When from the ages of doubt and care
Had science's form become more fair,
Her elements based on nature's worth,
Then *Hahnemann's* law was given birth;
Uniting first in science's form
The ills of life and poisons' harm,
A test he gave by which to know
How far in cure our powers go.

XI.

When nature in creative plan
Bade poison waste the life of man,
Our law of cure was given form,
To wait his time, its course to learn;
There nature made each poison's power
A guide unerring in suff'ring's hour;
The lines upon the stricken face
But point its use and mark its place.
Long ages dark o'er the world had sped,
While men taught views their own instead,
Reserved for *thee** the wond'rous view,
To see this law of *similars* true;

---

* *Hahnemann.*

It came, a beam of morning's light,
Across the fields of learning's night,
And as its ray falls o'er the field
The dream of the ages dark shall yield.

XII.

Now the reg'lars meet in long debate,
And gravely all their cures relate,
But heed you this, in candor true
Each treats his case from his own view,
While we who seek some common truth
Have found each "plan" devoid of proof;
The many "cures" of which we're told
Again prove true that law of old,
That nature would, if left alone,
Give many a cure which they now own,
While most the good they truly claim
But proves *similia* true again.

XIII.

Yes! men in vain their hours employ
Who would this law of cure destroy,
The blows they give must all rebound,
Their efforts topple to the ground;
Forget they must, that while they talk,
Our growing cause their doctrines balk,
That we, in nature's truth secure,
With nature must through time endure.

XIV.

Illusive plans and theories bold
Must go, as have mere *plans* of old;
No polished school, however strong,
Yet has proven *similia* wrong;
Their words to naught must come when tried
A poison's proving side by side;
Denials fall, devoid of harm,
Where nature's laws our cause affirm.

XV.

Unequal has the contest been;
'Tis nature opposed by plans of men—
The one, a *law* to grow in time,
Where better known the more sublime;
The other, changing views of men,
To be displaced till taught again.

XVI.

To those who read with bated breath,
And see in our growth science's death,
We would a word of comfort bring,
Nor yet the knell of science ring.

XVII.

All laws of science based on fact
Must through all time remain intact,

No idle whim or illusive plan,
Having its rise in mind of man,
Can stir from their inherent worth
The laws which have in nature birth.

XVIII.

We have but given her laws a test;
We ever *prove*, then use the best;
While men may glean in other fields,
Ours all its good in healing yields.
A century now almost has fled,
Its founder's sleeping with the dead,
Still, this great truth he taught, his race
Is growing on in science's place.
Co-teachers of his time and date
Taught views which seemed of greater weight,
But, as successive years have flown,
Theirs, with others, have grown and gone;
They had no therapeutic worth,
And left for science—medley's dearth.

XIX.

Why at the portals longer wait
When science opens wide her gate,
And bids us come, not, as before,
A struggling band, help to implore?
In honor now, despite the wrath
Of bigots who bar fair learning's path,
This mission of cure in all its range
Shall less and less true men estrange.

XX.

As we advance the healing art
The true and false must grow apart,
No common ground on which to meet—
One or the other must yet retreat;
Light and shadows can never blend,
Nor true and false to science tend;
'Tis nature bounds the wide domain
Where science e'er shall hold her reign;
In vain will men that bound define,
With narrow views of their own time.

XXI.

Our art but grows from nature's laws,
We learn the fact, then trace its cause;
Long had the heart the pulses made,
Long at the pulse men had stayed,
Till Harvey's mind, with vision keen,
Traced from this fact its cause unseen.
So Hahnemann's law* of nature's cure
Existed ere his natal hour;
He but traced the subtle truth
Back to nature, there found its proof;
So we who would this truth proclaim
Will ever find its law the same.

EDWARD B. ATKINS.

* *Similia similibus curantur.*

